# Effects of the BOPPPS model combined with case-based learning on knowledge acquisition and learning engagement in undergraduate nursing students: a multi-cohort quasi-experimental study

**DOI:** 10.1186/s12909-026-09428-9

**Published:** 2026-05-23

**Authors:** Yuda Fei, Yaning Zhang, Jie Cai, Mei Xu, Jing Huang, Bin Du

**Affiliations:** 1 Department of Infection Control, Peking Union Medical College Hospital, Chinese Academy of Medical Sciences & Peking Union Medical College, Beijing, China; 2 Department of Medical ICU, State Key Laboratory of Complex Severe and Rare Diseases, Peking Union Medical College Hospital, Chinese Academy of Medical Sciences & Peking Union Medical College, Beijing, China

**Keywords:** BOPPPS model, Case-Based Learning (CBL), Healthcare-Associated Infection (HAI), Nursing Education, Quasi-Experiment, Historical Control, Problem-Based Learning, Infection Control, Students, Nursing

## Abstract

**Background:**

Healthcare-Associated Infection (HAI) prevention is a crucial competency in nursing education but is often taught through didactic lectures with limited application. This study evaluated the effectiveness of combining the Bridge-in, Objective, Pre-assessment, Participatory Learning, Post-assessment, and Summary (BOPPPS) model with Case-Based Learning (CBL) for HAI education using a non-equivalent control group design.

**Methods:**

A quasi-experimental study with a historical control group was conducted over three consecutive academic years (2023–2025). Second-year nursing undergraduates enrolled in an elective HAI module participated. The 2023 cohort (control group, *n* = 21) received traditional lecture-based learning (LBL). The 2024 (*n* = 18) and 2025 (*n* = 25) cohorts (intervention group, total *n* = 43) received instruction redesigned using the BOPPPS framework with CBL. Knowledge acquisition was assessed via identical pre- and post-course tests, analyzed using analysis of covariance (ANCOVA) with pre-test scores as covariates. Post-course perceptions were measured using an 8-dimension, 5-point Likert-scale questionnaire. Data were analyzed following the Strengthening the Reporting of Observational Studies in Epidemiology (STROBE) guidelines adapted for educational interventions.

**Results:**

Baseline characteristics were comparable (pre-test score: LBL = 54.00 ± 6.49 vs. BOPPPS-CBL = 52.30 ± 7.11, *p* = 0.360, Cohen’s d = 0.17). ANCOVA revealed that the BOPPPS-CBL group achieved significantly higher post-test scores after adjusting for baseline differences (adjusted mean = 87.19 vs. 73.61; mean difference = 13.58, 95% CI: 11.14–16.03, F(1,61) = 123.46, *p* < 0.001, partial eta-squared = 0.669). Within-group improvements were significant for both conditions (both *p* < 0.001). The intervention group reported significantly higher scores across all seven positive perception dimensions (all *p* < 0.001 after Bonferroni correction), including self-directed learning (4.07 ± 0.80 vs. 3.19 ± 0.68, Cohen’s d = 1.15) and teacher-student interaction (3.98 ± 0.80 vs. 2.76 ± 0.94, Cohen’s d = 1.43). Notably, they also reported higher perceived learning burden (3.98 ± 0.86 vs. 2.29 ± 0.96, *p* < 0.001, Cohen’s d = 1.90). Sensitivity analyses showed consistent intervention effects across the 2024 and 2025 cohorts (cohort-by-interaction *p* = 0.737). Qualitative feedback highlighted enhanced knowledge application and autonomous learning skills.

**Conclusions:**

The BOPPPS-CBL model appears to be effective in enhancing HAI knowledge acquisition and fostering positive learning engagement among undergraduate nursing students. The increased perceived cognitive effort may represent ‘productive cognitive engagement’ rather than ‘productive germane load’. This structured, active learning strategy offers a promising approach for teaching critical infection control competencies, though resource implications and design limitations warrant consideration. Institutions adopting this model should ensure rigorous case design and faculty development.

**Supplementary Information:**

The online version contains supplementary material available at 10.1186/s12909-026-09428-9.

## Introduction

Healthcare-Associated Infections (HAIs) pose a significant threat to patient safety and represent a core focus of clinical practice and nursing education [1]. Nurses play a pivotal role in HAI prevention through direct patient care and infection control protocol implementation. However, educational approaches in undergraduate curricula are often fragmented and rely heavily on passive knowledge transmission, limiting students’ ability to apply theoretical knowledge in complex clinical contexts [2]. This challenge involves multiple stakeholders, including educational institutions, policy makers, and accreditation bodies, highlighting the need for systemic pedagogical reform.

International studies from diverse settings consistently highlight gaps in nurses’ and students’ knowledge regarding infection prevention and control [3–5]. In Ghana, critical deficiencies were identified in standard precaution practices among nursing staff [3], while research in Sri Lanka revealed suboptimal knowledge of nosocomial infections among physiotherapy undergraduates [5]. These findings, while context-specific, underscore a universal challenge in translating infection control knowledge into consistent clinical practice across different healthcare systems.

The BOPPPS model-a structured, student-centered instructional framework comprising Bridge-in, Objective, Pre-assessment, Participatory Learning, Post-assessment, and Summary-promotes active learning and continuous feedback [6]. Grounded in constructivist learning theory, BOPPPS emphasizes learner engagement and scaffolding. Case-Based Learning (CBL) contextualizes theoretical knowledge within authentic clinical narratives, enhancing clinical reasoning and problem-solving capabilities [7]. Their integration (BOPPPS-CBL) has demonstrated efficacy across various medical education domains, including ECG interpretation for nursing students [8], ophthalmology [9], and thoracic surgery [10], consistently producing improved knowledge scores and learner satisfaction compared to traditional Lecture-Based Learning (LBL). A systematic review and meta-analysis confirmed the superior effectiveness of BOPPPS-based strategies in Chinese medical education [11], while a recent network meta-analysis identified CBL as one of the most effective novel strategies for improving theoretical scores in medical education [12].

However, the application of this integrated model in the specific and critical domain of HAI education for undergraduate nurses remains underexplored. Critically, existing evaluations of BOPPPS-based strategies predominantly rely on immediate post-intervention assessments using non-validated, study-specific tests, with little evidence of sustained knowledge retention or transfer to clinical practice. Moreover, the majority of studies originate from single institutions in China, limiting the generalizability of their findings to other educational and cultural contexts. HAI prevention represents a protocol-driven, high-stakes domain where errors have immediate patient safety consequences, distinguishing it from the diagnostic skills (e.g., ECG interpretation) examined in previous BOPPPS-CBL studies. Furthermore, existing evaluations primarily rely on immediate post-intervention assessments (Kirkpatrick Level 2) [13], with limited evidence regarding knowledge retention or clinical behavior transfer (Kirkpatrick Levels 3–4).

While recent studies have called for enhanced educational models for infection preventionists [14] and explored simulation-based learning [15], there is a paucity of research evaluating structured, active learning frameworks like BOPPPS-CBL for foundational HAI training in pre-licensure nursing programs. This study aimed to bridge this gap by evaluating the effects of the BOPPPS-CBL model on knowledge acquisition and learning engagement in HAI education over three academic years using a non-equivalent control group design.Specifically, we sought to determine whether the BOPPPS-CBL approach produces superior learning outcomes compared to traditional lecture-based instruction in this critical competency domain.

## Methods

### Study design

This quasi-experimental study employed a non-equivalent control group design with a historical comparison cohort, conducted over three consecutive academic years (2023–2025) at Peking Union Medical College, China. We followed the Strengthening the Reporting of Observational Studies in Epidemiology (STROBE) guidelines for observational studies [16], adapted for this educational intervention context. Although STROBE is designed for observational studies, it was selected because our quasi-experimental design with a historical control shares key validity threats (e.g., selection bias, history effects) with observational research. A dedicated educational reporting guideline such as TREND (Transparent Reporting of Evaluations with Nonrandomized Designs) was considered, but STROBE was chosen for consistency with our institutional protocol. We acknowledge that future educational quasi-experiments may benefit from using TREND.

The study participants comprised second-year undergraduate nursing students enrolled in the elective module “Nosocomial Infection and Prevention” at the School of Nursing. The 2023 cohort served as the historical control group, taught using the lecture-based learning (LBL) method (*n* = 21), while the 2024 (*n* = 18) and 2025 (*n* = 25) cohorts constituted the intervention group, instructed via the BOPPPS-CBL pedagogical approach (total *n* = 43).

### Sample size and power analysis

A prospective sample size calculation was performed a priori using G*Power 3.1.9.7. Based on prior BOPPPS-CBL studies in nursing education reporting effect sizes ranging from d = 0.6 to d = 1.0 [8], we conducted sensitivity analyses across a range of plausible effect sizes. With alpha = 0.05 (two-tailed) and power = 1-beta = 0.80, the required sample sizes were: d = 0.5: *n* = 64 per group; d = 0.6: *n* = 45 per group; d = 0.8: *n* = 25 per group; d = 1.0: *n* = 16 per group. Our achieved sample (LBL: *n* = 21; BOPPPS-CBL: *n* = 43) provides adequate power (> 0.80) to detect medium-to-large effects (d > = 0.65). We emphasize effect sizes and confidence intervals rather than post-hoc power in our interpretation, as recommended [17]. We acknowledge that the small sample size and inequality between groups may impact statistical precision, and we have interpreted findings with appropriate caution. Specifically, the control group (*n* = 21) and intervention group (*n* = 43) are unequal, which may reduce the precision of the ANCOVA estimates and increase the risk of Type II error for detecting small-to-medium effects. We therefore emphasize effect sizes and confidence intervals rather than p-values alone.

### Participants and recruitment

**Fig. 1:**
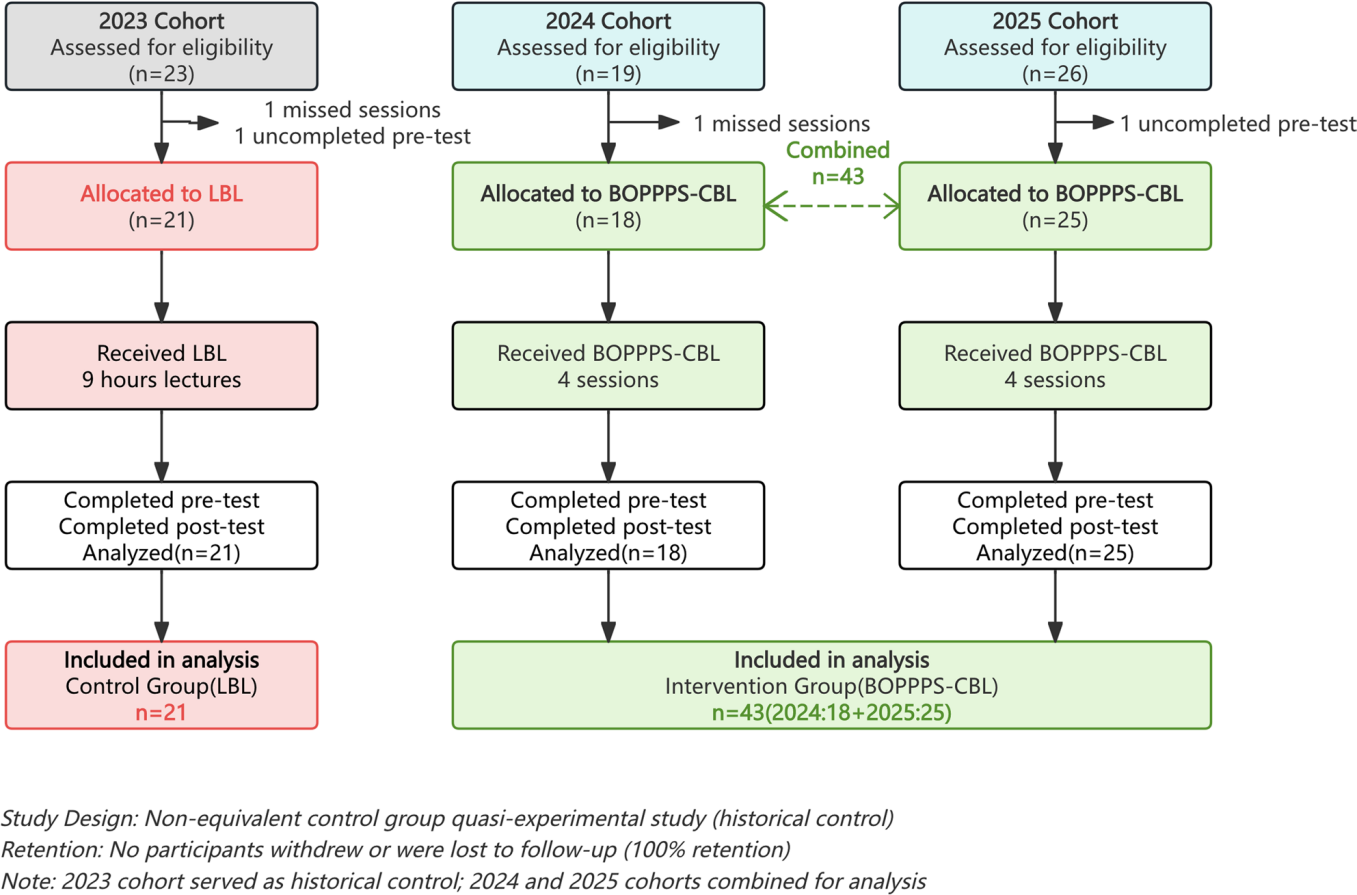
Participant flow diagram through the multi-cohort study. Flowchart showing enrollment, group assignment, and retention of participants across the 2023 (control), 2024 (intervention), and 2025 (intervention) cohorts. All enrolled students completed the study (retention rate: 100%)

Total eligible students per cohort were: 2023 (*n* = 23), 2024 (*n* = 19), and 2025 (*n* = 26). Participation rates were high across all cohorts: 91.3% (2023), 94.7% (2024), and 96.2% (2025). Inclusion criteria were: (1) enrolled second-year nursing students registered in the HAI module, and (2) completion of both pre- and post-tests. Exclusion criteria were: (1) missing more than one teaching session, or (2) incomplete test data. All enrolled students completed the study (retention rate: 100%). Figure 1 presents the participant flow diagram.

### Control of confounding variables

To isolate the effect of the teaching model and minimize threats to internal validity, rigorous consistency controls were implemented (Table 1). Key threats and mitigation strategies included:

**Table 1:** Threats to internal validity and mitigation strategies

Threat	Description	Mitigation Strategy	Residual Risk
History	External events (e.g., COVID-19 pandemic) may differentially affect cohorts	Same academic term timing; documented timeline of pandemic phases; sensitivity analysis across cohorts	High
Maturation	Natural changes in students over time independent of intervention	All participants were second-year students with comparable prior coursework	Low
Selection	Systematic differences between cohorts in motivation or ability	Elective module with high participation rates (> 91%); comparable baseline characteristics	Low
Instrumentation	Changes in measurement procedures or grading standards across years	Identical test items and scoring rubric across all cohorts; standardized procedures	Low
Testing	Practice effects from repeated testing with identical instruments	Both groups experienced identical testing conditions; between-group comparison remains valid	Moderate
Regression to Mean	Extreme scores tend to move toward the mean on retesting	ANCOVA used to control for baseline differences; effect sizes reported	Low

*ANCOVA* Analysis of Covariance, *LBL* Lecture-Based Learning, *BOPPPS*-*CBL* Bridge-in, Objective, Pre-assessment, Participatory Learning, Post-assessment, and Summary combined with Case-Based Learning

Instructional Consistency: The same faculty team (two infection control physicians and one senior infection control nurse) with expertise in infection control and nursing education taught all cohorts. Teaching evaluations (student ratings) confirmed comparable instructor effectiveness across years (mean scores: 4.6/5.0 for 2023; 4.7/5.0 for 2024; 4.6/5.0 for 2025; *p* = 0.412). However, we acknowledge that instructor equivalence does not ensure instructional equivalence, as pedagogy changed substantially between conditions.

Content Consistency: The core syllabus, total contact hours (9 h), core HAI knowledge and skill objectives, assignment requirements (one reflective essay on infection control practices), and the scope/content of the final knowledge assessment were held identical across conditions. The same instructors delivered both LBL and BOPPPS-CBL instruction, which may introduce instructor-related effects. While we standardized instructor training for the BOPPPS-CBL intervention, we cannot rule out the possibility that instructor enthusiasm or implementation quality differed between conditions.

Temporal Consistency: All sessions for both groups were conducted during the same academic term (semester 1, year 2) and at comparable times of day (evening sessions: 7:00–9:00 PM) to minimize circadian variation effects. 2023 cohort (control) taught in Autumn Semester 2023 (September-December 2023), 2024 and 2025 cohorts (intervention) taught in Autumn Semesters 2024 and 2025。.

Student Maturity: All participants were second-year students with comparable prior academic coursework (completed microbiology and basic nursing principles).

### Intervention development and description

#### Instructor training and fidelity monitoring

Instructors delivering the BOPPPS-CBL intervention completed an 8-hour training workshop covering BOPPPS framework principles, facilitative teaching techniques, and Socratic questioning methods. They also conducted practice sessions with peer feedback before implementation.

Intervention fidelity was monitored using a standardized BOPPPS implementation checklist completed by independent observers for 20% of sessions (randomly selected). The checklist assessed completion of all six BOPPPS components within specified time allocations and quality of facilitation. Mean fidelity score was 94.2% (SD = 4.8%) across all observed sessions, indicating high adherence to the intervention protocol.

#### CBL case development and validation

Four core CBL cases were developed for the participatory learning phases, adapted from anonymized, real HAI event reports within our hospital’s infection control database (2020–2022), ensuring clinical authenticity. Each case targeted specific curriculum competencies: (1) Central-line-associated bloodstream infection (CLABSI) prevention bundle violations; (2) Catheter-associated urinary tract infection (CAUTI) and aseptic technique; (3) Ventilator-associated pneumonia (VAP) and multi-modal prevention strategies; and (4) Multi-drug resistant organism (MDRO) transmission via surgical site infection. Cases included relevant patient history, clinical data (laboratory results, vital signs), and targeted questions prompting identification of breach points in prevention bundles, root cause analysis, and formulation of corrective actions.

Content validity was established through expert review by a panel comprising two infection control physicians and two senior nursing education specialists. The item-level Content Validity Index (I-CVI) ranged from 0.83 to 1.00, and the scale-level CVI (S-CVI/Ave) was 0.94, indicating excellent content validity [18]. A pilot test with 12 senior nursing students (not involved in the main study) assessed case flow, clarity, and time requirements, leading to minor adjustments in terminology.

#### BOPPPS-CBL intervention protocol (Fig. 2)

**Fig. 2:**
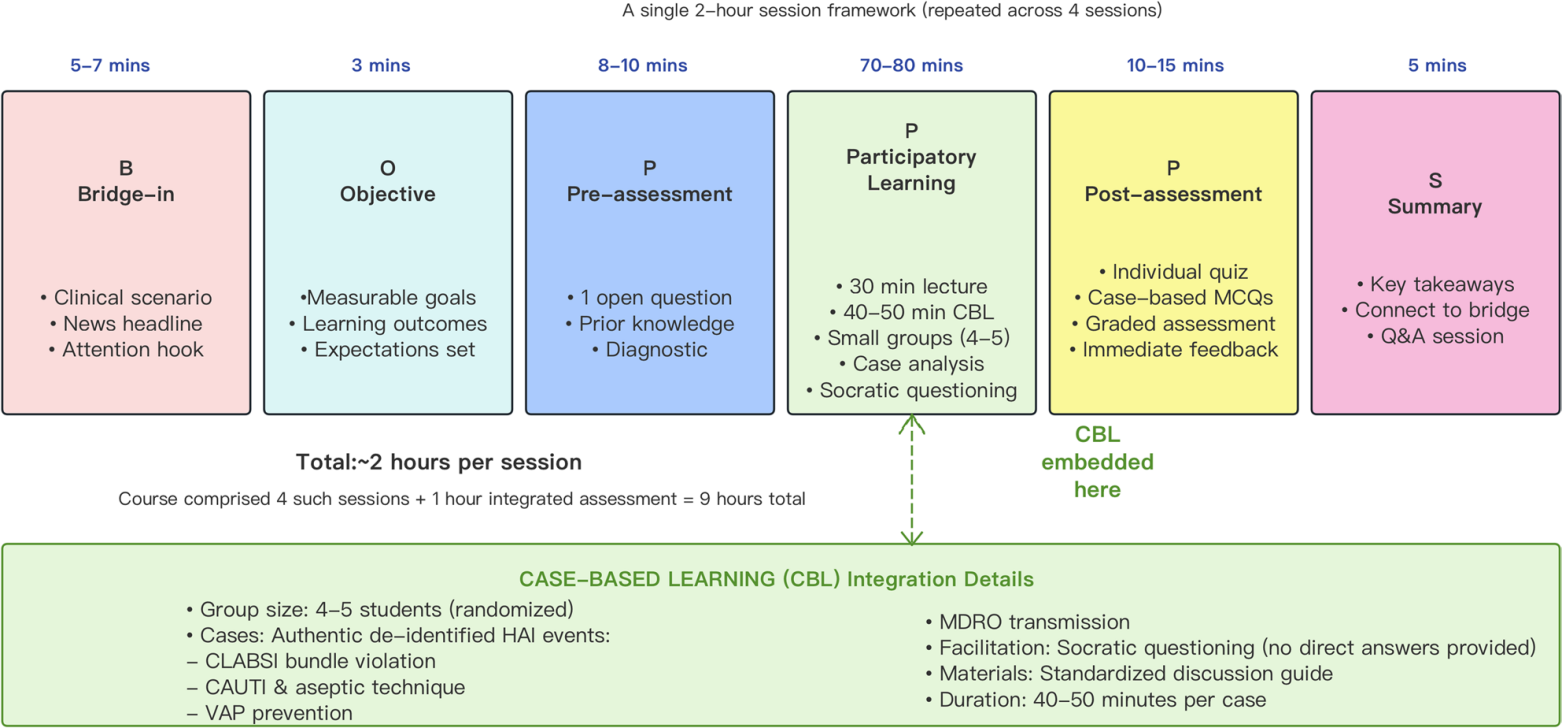
The BOPPPS-CBL instructional model structure

The 9-hour course was structured into four 2-hour sessions plus a 1-hour integrated assessment session, each following the BOPPPS framework with CBL integrated into the Participatory Learning phase. The Bridge-in component (5–7 min) began each session with an impactful clinical scenario or recent HAI outbreak news headline to capture attention. The Objective component (3 min) presented clear, measurable learning objectives. The Pre-assessment (8–10 min) used a short, non-graded online assessment with 5 multiple-choice questions testing foundational knowledge and 1 open-ended question to gauge prior conceptions. Participatory Learning (70–80 min) combined a 30-minute concise didactic lecture with a 40–50 min structured CBL activity where students worked in small groups (4–5 students) to analyze cases using a standardized discussion guide, with instructors facilitating through Socratic questioning. Post-assessment (10–15 min) involved individual, graded case-based assessments linked to session objectives. The Summary component (5 min) reviewed key points and connected them to the bridge-in scenario and objectives.

#### Control group (LBL) protocol

The control group received the same core content delivered via 9 h of traditional didactic lectures (7.5 h) supplemented with instructor-led question-and-answer sessions (1.5 h). No structured small-group CBL activities or pre/post-assessments were conducted.

### Outcome measures and tools

#### Primary outcome: knowledge acquisition

Knowledge was assessed using a self-developed, validated 20-item multiple-choice question (MCQ) test (total score 100 points) covering core HAI topics. The test was developed by two infection control managers and one senior infection control nurse with expertise in nursing education. Test items were mapped to four content domains: transmission routes (5 items), standard precautions (6 items), device-related infections (5 items), and antimicrobial stewardship (4 items). Content validity was established (S-CVI/Ave = 0.92) through expert review. Internal consistency was adequate (Cronbach’s alpha = 0.81 for post-test data). Item analysis revealed appropriate difficulty indices (0.45–0.75) and discrimination indices (0.30–0.55) for all items.

Identical test items were used for pre- and post-tests across all three cohorts (2023–2025). Test security was maintained through in-person administration, collection of all materials after testing, and secure server storage. No electronic copies were distributed to students. Two independent graders scored short-answer items using a standardized rubric for 20% of tests, with inter-rater reliability of Cohen’s kappa = 0.89. Graders were blinded to group assignment during scoring.

We acknowledged that employing identical instruments for pre- and post-testing may introduce testing effects, such as recall or familiarity, which could artificially inflate the measured learning gains. However, as both experimental groups were exposed to the same testing conditions, the validity of between-group comparisons is preserved. This aspect is addressed as a methodological limitation. The substantial effect sizes observed (e.g., Cohen’s d = 1.77 for gain scores) may be partially attributable to this familiarity effect, alongside the genuine pedagogical impact. Thus, readers should interpret the magnitude of learning gains with this caveat in consideration.

#### Secondary outcome: student perceptions

Learning engagement and perceptions were measured using a post-course questionnaire adapted from validated instruments [8, 11]. Exploratory Factor Analysis (EFA) supported a 7-factor structure for positive perceptions: Self-directed Learning, Systematic Content Organization, Depth of Understanding, Teacher-Student Interaction, Satisfaction, Perceived Effectiveness, and Interest in Learning. One additional item assessed Perceived Learning Burden (not reverse-coded-higher scores indicate greater perceived burden).The full questionnaire, including all items and their factor loadings, is provided as Supplementary Table S2.

The KMO measure of sampling adequacy was 0.88, and Bartlett’s test of sphericity indicated sufficient correlations (chi-square = 892.4, *p* < 0.001). Internal consistency was excellent (Cronbach’s alpha = 0.89 for the 7 positive dimensions). The factor loadings for each item ranged from 0.62 to 0.85, supporting the 7-factor structure. However, the EFA was conducted with a combined sample of *N* = 64, which is relatively small for factor analysis; the stability of the factor structure should be confirmed in larger samples in future studies. The full questionnaire, including all items and their factor loadings, is provided as Supplementary Table S2. Responses were recorded on a 5-point Likert scale (1 = Strongly Disagree to 5 = Strongly Agree). We acknowledge that EFA with *N* = 64 is exploratory; factor structure should be confirmed with larger samples. Likert scales were treated as continuous variables based on methodological evidence supporting this approach for scales with 5 + points [19].

#### Qualitative feedback

Open-ended questions explored students’ experiences with the learning model: ‘What specific aspects of the case discussions helped or hindered your learning?’ and ‘How has this course format influenced your approach to studying infection control?’ These BOPPPS-specific questions were ONLY included in 2024/2025 evaluations (intervention cohorts). The 2023 control group had standard course evaluation without BOPPPS-specific probes, limiting qualitative comparability between groups. This introduces a systematic measurement bias where the student voice is only heard from the group receiving the new intervention.

Two research assistants (who were graduate students in health education affiliated with the institution, not course instructors) performed independent thematic analysis in accordance with the methodology outlined by Braun and Clarke [20]. They were blinded to quantitative outcomes during analysis. Initial independent coding was followed by comparison, discussion to consensus, and consultation with a third researcher when needed. Inter-rater reliability for double-coded responses (20%) was Cohen’s kappa = 0.82. Thematic saturation was assessed by examining whether new themes emerged in the final 5 responses; no new themes were identified, suggesting saturation was achieved.

### Statistical analysis

Data were analyzed using R software (version 4.5.2). Normality was confirmed via Shapiro-Wilk test (all *p* > 0.05).

Primary Analysis: Analysis of Covariance (ANCOVA) was used to compare post-test scores between groups, with pre-test scores entered as covariates to control for baseline differences [21]. ANCOVA assumptions were tested: (1) Linearity: Scatterplots of pre-test versus post-test scores within each group supported linear relationships, with no significant correlations found in either the control (*r* = − 0.01, *p* = 0.966) or intervention group (*r* = − 0.17, *p* = 0.272). The weak negative correlations likely reflect greater improvement among students with lower baseline knowledge, which does not violate the ANCOVA linearity assumption as it primarily requires linearity in form rather than strength or direction; (2) Homogeneity of regression slopes: non-significant group x pre-test interaction (F(1,60) = 0.42, *p* = 0.521); (3) Normality of residuals: Shapiro-Wilk test *p* = 0.312; (4) Homoscedasticity: Levene’s test *p* = 0.487. All assumptions were met. Partial eta-squared was reported as effect size (small = 0.01, medium = 0.06, large = 0.14).

Secondary Analyses: Within-group pre-post comparisons used paired samples t-tests. Between-group comparisons of questionnaire data used independent samples t-tests and Mann-Whitney U tests as non-parametric alternatives. Given multiple comparisons (8 dimensions), Bonferroni correction was applied (adjusted alpha = 0.006). Cohen’s d was calculated for all comparisons as effect size measure. Sensitivity analyses compared the 2024 and 2025 intervention subgroups against the control group using one-way ANCOVA, testing for cohort-by-intervention interactions.

Qualitative Data: Thematic analysis (Braun and Clarke) [20] was used with double-coding of 20% of responses (Cohen’s kappa = 0.82).

Two-tailed p-values < 0.05 were considered statistically significant for primary outcomes; <0.006 for secondary outcomes.

## Results

### Baseline characteristics

Baseline characteristics were comparable between the historical control (LBL) and intervention (BOPPPS-CBL) groups (Table 2). No significant differences were observed in age (*p* = 0.663, Cohen’s d = 0.12), gender distribution (*p* = 0.938, Cramer’s V = 0.01), or pre-test knowledge scores (*p* = 0.360, Cohen’s d = 0.25). The intervention group showed slightly lower (non-significant) baseline scores (52.30 ± 7.11 vs. 54.00 ± 6.49). We acknowledge that the small sample size may limit the power to detect relevant initial differences, especially considering the tendency for slightly lower pre-test scores in the intervention group. For example, the observed baseline difference of -1.70 points (95% CI: -4.96 to 1.56) could represent either a true slight disadvantage of the intervention group or random sampling variation; ANCOVA was used to statistically adjust for this remaining imbalance.

**Table 2:** Baseline characteristics of participants

Characteristic	Control (LBL) *n* = 21	Intervention (BOPPPS-CBL) *n* = 43	Statistical Test	*p*-value	Effect Size
Age, years (Mean ± SD)	19.14 ± 0.85	19.05 ± 0.82	Independent t-test	0.663	Cohen’s d = 0.12
Gender, Female n (%)	18 (85.7)	35 (81.4)	Chi-square test	0.938	Cramer’s V = 0.01
Pre-test Score (Mean ± SD)	54.00 ± 6.49	52.30 ± 7.11	Independent t-test	0.360	Cohen’s d = 0.25

Data are presented as Mean ± SD for continuous variables and n (%) for categorical variables. Cohen’s d interpreted as: negligible (< 0.2), small (0.2–0.5), medium (0.5–0.8), large (> 0.8). Cramer’s V interpreted as: small (0.1–0.3), medium (0.3–0.5), large (> 0.5)

*LBL* Lecture-Based Learning, *BOPPPS*-*CBL* Bridge-in, Objective, Pre-assessment, Participatory Learning, Post-assessment, and Summary combined with Case-Based Learning

### Knowledge acquisition

Both groups demonstrated significant within-group knowledge improvement from pre-test to post-test (both *p* < 0.001). However, the magnitude of improvement differed substantially between conditions.

**Fig. 3:**
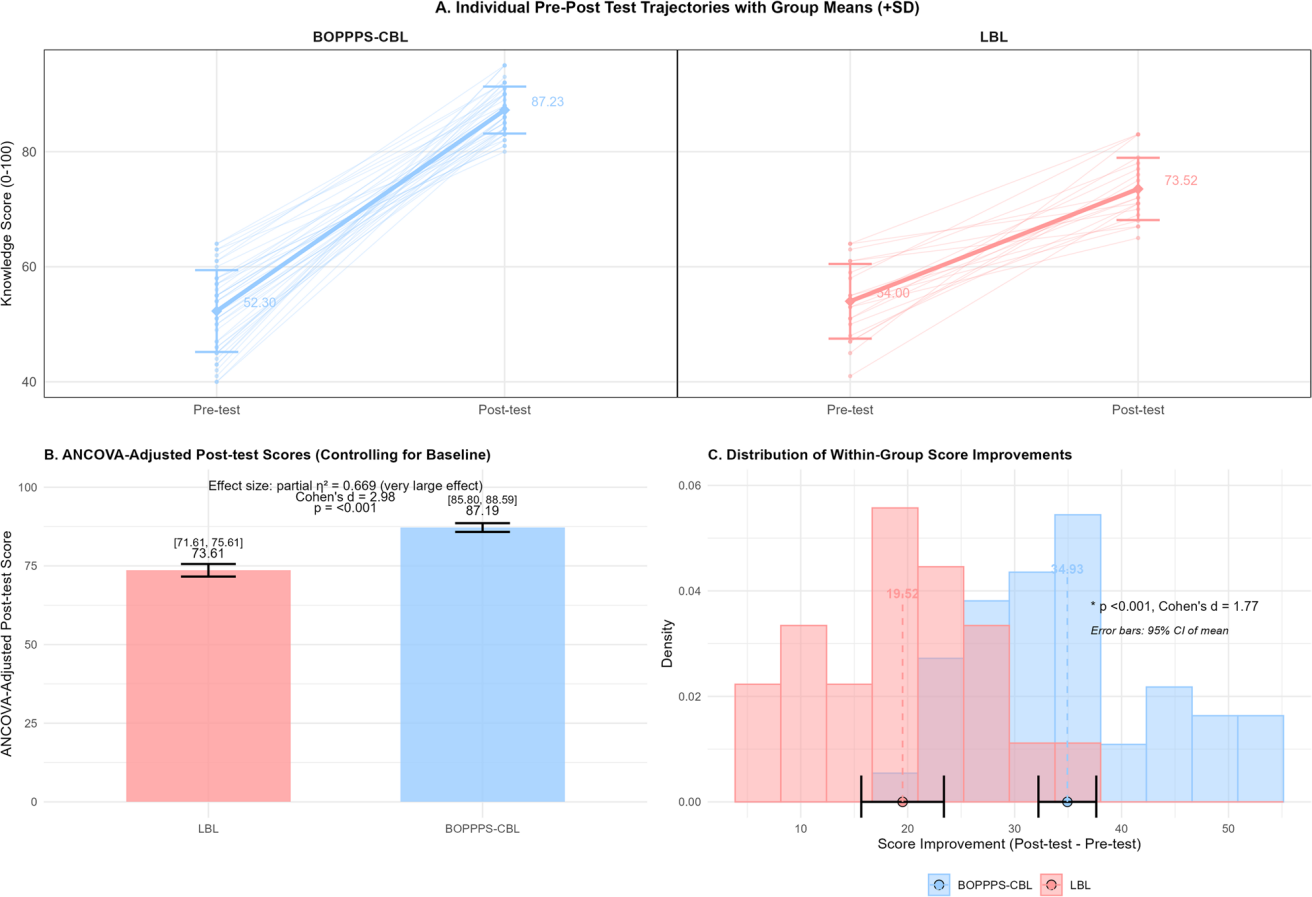
Comparison of knowledge scores (Mean +/- 95% CI). **A** Unadjusted Post-test Scores: BOPPPS-CBL group scored significantly higher than LBL group (*p* < 0.001). **B** ANCOVA-Adjusted Post-test Scores: Adjusted for baseline pre-test differences, showing maintained significant advantage for BOPPPS-CBL (adjusted mean difference = 13.58, 95% CI: 11.14–16.03, *p* < 0.001, partial eta-squared = 0.669). **C** Score Improvement (Post-pre): BOPPPS-CBL group showed significantly greater improvement than LBL group (*p* < 0.001, Cohen’s d = 1.77). Note: *p < 0.001; error bars represent 95% confidence intervals

ANCOVA controlling for baseline pre-test scores revealed that the BOPPPS-CBL group achieved significantly higher post-test scores compared to the LBL group (adjusted mean difference = 13.58, 95% CI: 11.14–16.03, F(1,61) = 123.46, *p* < 0.001). The effect size was large (partial eta-squared = 0.67), indicating that 67% of the variance in post-test scores was explained by the potential teaching-to-the-test effects or ceiling effects in the intervention group after controlling for baseline knowledge (Table 3; Fig. 3).

**Table 3:** Comparison of knowledge acquisition outcomes (ANCOVA and t-test Results)

Outcome	Control (LBL)	Intervention (BOPPPS-CBL)	Mean Difference [95% CI]	Cohen’s d	t / F-value	*p*-value	eta-squared-*p*
Pre-test Score	54.00 (6.49)	52.30 (7.11)	-1.70 [-4.96, 1.56]	0.25	t(62) = 0.92	0.360	0.014
Post-test (Unadjusted)	73.52 (5.41)	87.23 (4.08)	13.71 [11.26, 16.16]	3.01	t(62) = 11.31	< 0.001	0.674
Post-test (Adjusted)	73.61 (1.00) [71.6, 75.6]	87.19 (0.70) [85.8, 88.6]	13.58 [11.14, 16.03]	2.98	F(1,61) = 123.46	< 0.001	0.669
Score Improvement	19.52 (8.49)	34.93 (8.78)	15.41 [10.78, 20.03]	1.77	t(62) = 6.66	< 0.001	0.417

Pre-test comparisons used independent samples t-tests. Post-test (Adjusted) comparison used ANCOVA with pre-test as covariate. Score Improvement comparison used independent samples t-test on gain scores. Values in parentheses for adjusted scores are standard errors of the mean (SE); values for unadjusted scores are standard deviations (SD). Partial eta-squared interpreted as: small (0.01), medium (0.06), large (0.14)

*ANCOVA* Analysis of Covariance, *CI* Confidence Interval, *LBL* Lecture-Based Learning; BOPPPS-CBL = Bridge-in, Objective, Pre-assessment, Participatory Learning, Post-assessment, and Summary combined with Case-Based Learning.

Within-group improvements were 19.52 points (95% CI: 15.66–23.39) for LBL and 34.93 points (95% CI: 32.23–37.63) for BOPPPS-CBL. Gain score analysis confirmed significantly greater improvement in the intervention group (mean difference = 15.41, 95% CI: 10.78–20.03, *p* < 0.001, Cohen’s d = 1.77).

We acknowledge that the effect sizes are unusually large for educational interventions. Potential contributing factors include: (1) the protocol-driven nature of HAI content with clear correct answers for bundle compliance; (2) test alignment with learning objectives; (3) intensive small-group facilitation; (4) novelty effect of active learning for students accustomed to lectures; and (5) possible testing effects from identical pre/post tests. These large effects may be context-specific and may not generalize to all educational settings. It is imperative to acknowledge that the exceptionally large statistical effect sizes may be partially attributable to the utilization of identical pre- and post-test instruments, which can inflate perceived learning gains due to familiarity effects. Furthermore, these elevated effect sizes may indicate teaching-to-the-test effects or ceiling effects within the intervention group, wherein rigorous preparation for case-based assessments resulted in enhanced performance on the specific test items employed. This constitutes a crucial consideration for readers endeavoring to replicate the model across diverse nursing contexts.

### Cohort sensitivity analysis

**Fig. 4:**
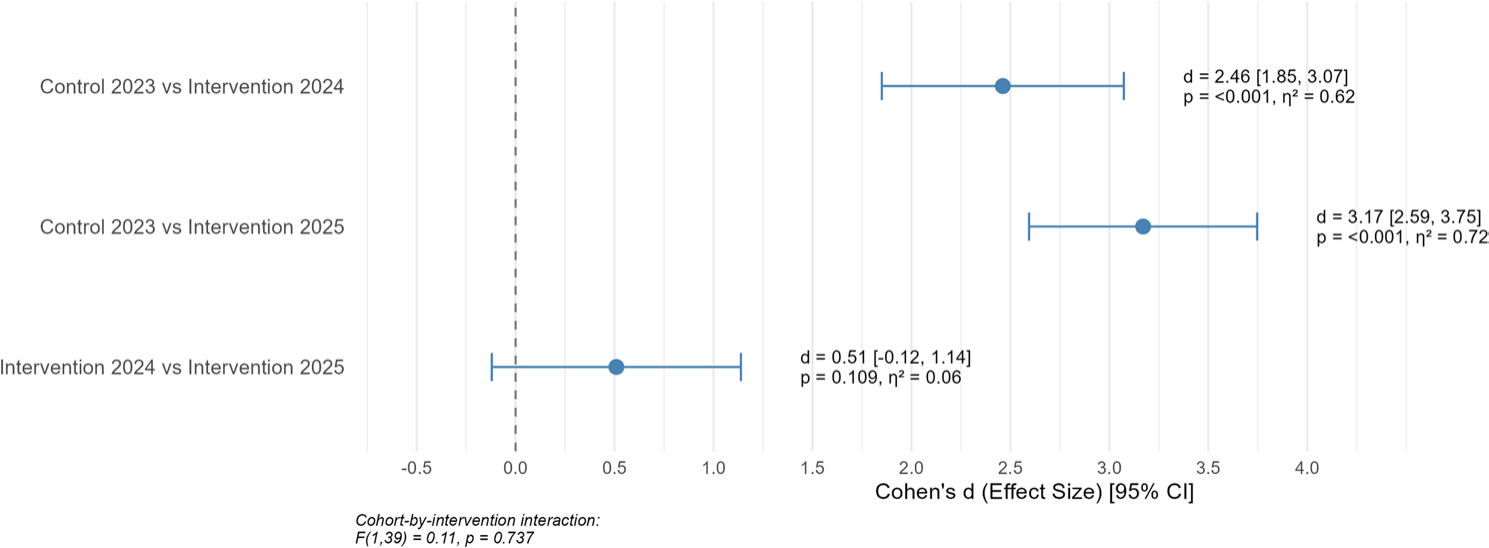
Forest Plot of Intervention Effects by Cohort. Forest plot showing effect sizes (Cohen’s d) and 95% confidence intervals for knowledge acquisition outcomes comparing each intervention cohort (2024, 2025) to the control cohort (2023). The cohort-by-intervention interaction was non-significant (*p* = 0.737), indicating consistent effects across intervention years

Comparison of the 2024 (*n* = 18) and 2025 (*n* = 25) intervention subgroups revealed no significant differences in post-test scores after controlling for baseline (adjusted mean:86.05 vs. 88.08, *p* = 0.109). The cohort-by-intervention interaction was non-significant (F(1,39) = 0.11, *p* = 0.737), confirming consistency of the intervention effect across years (Fig. 4; Table 4).

**Table 4:** Sensitivity analysis: cohort-specific intervention effects

Comparison	Adjusted Mean (SE)	Mean Difference [95% CI]	F-value	*p*-value	eta-squared-*p*
Control 2023 vs. Intervention 2024	73.61 (1.00) vs. 86.05 (1.05)	12.44 [9.35, 15.53]	57.69	< 0.001	0.62
Control 2023 vs. Intervention 2025	73.61 (1.00) vs. 88.08 (0.89)	14.47 [11.84, 17.10]	112.63	< 0.001	0.72
Intervention 2024 vs. Intervention 2025	86.05 (1.05) vs. 88.08 (0.89)	2.03 [-0.48, 4.54]	2.69	0.109	0.06

Cohort-by-intervention interaction test: F(1,39) = 0.11, *p* = 0.737, indicating consistent intervention effects across 2024 and 2025 cohorts. Effect sizes calculated from ANCOVA-adjusted means with pre-test as covariate

*ANCOVA* Analysis of Covariance, *CI* Confidence Interval, *SE* Standard Error

### Student perceptions and learning engagement

**Fig. 5:**
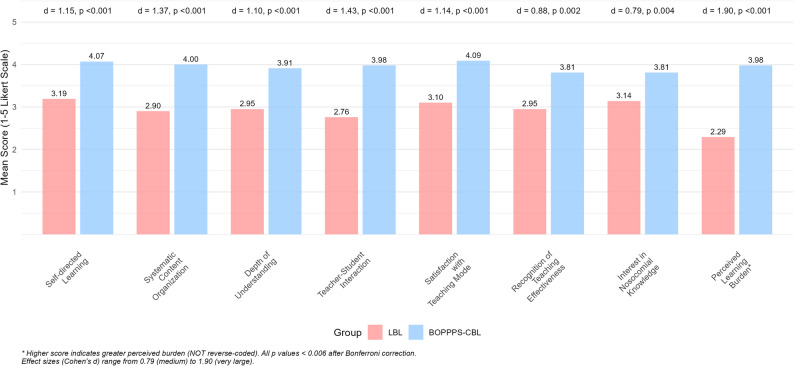
Comparison of Mean Scores on Post-course Perception Dimensions Between Groups. Bar chart illustrating mean scores for seven positive perception dimensions and Perceived Learning Burden. The BOPPPS-CBL group (blue bars) scored higher on all dimensions. Note that Perceived Learning Burden is not reverse-coded-higher scores indicate greater perceived burden. Y-axis represents Likert scale scores (1–5). ***p < 0.001 after Bonferroni correction. Effect sizes (Cohen’s d) ranged from 0.79 to 1.90

The BOPPPS-CBL group reported significantly higher scores across all seven positive perception dimensions after Bonferroni correction (all *p* < 0.006), including key learning engagement indicators: Teacher-Student Interaction (3.98 ± 0.80 vs. 2.76 ± 0.94; mean difference = 1.21, 95% CI: 0.76–1.67, Cohen’s d = 1.43) and systematic content organization 4.00 ± 0.82 vs. 2.90 ± 0.77; mean difference = 1.10, 95% CI: 0.67–1.52, Cohen’s d = 1.37) (Table 5; Fig. 5). Non-parametric Mann-Whitney U tests yielded consistent results with parametric t-tests (all *p* < 0.001; see Supplementary Table S1).

**Table 5:** Comparison of survey dimensions between control and intervention groups(*n* = 64)

Survey Dimension (1–5 Likert)	Control (LBL)	Intervention (BOPPPS-CBL)	Mean Difference [95% CI]	Cohen’s d	Effect Size	*p*-value
Self-directed Learning	3.19 ± 0.68	4.07 ± 0.80	0.88 [0.47, 1.28]	1.15	Large	< 0.001
Systematic Content Organization	2.90 ± 0.77	4.00 ± 0.82	1.10 [0.67, 1.52]	1.37	Large	< 0.001
Depth of Understanding	2.95 ± 0.80	3.91 ± 0.89	0.95 [0.49, 1.42]	1.10	Large	< 0.001
Teacher-Student Interaction	2.76 ± 0.94	3.98 ± 0.80	1.21 [0.76, 1.67]	1.43	Large	< 0.001
Satisfaction with Teaching Mode	3.10 ± 1.00	4.09 ± 0.81	1.00 [0.53, 1.46]	1.14	Large	< 0.001
Recognition of Teaching Effectiveness	2.95 ± 1.20	3.81 ± 0.85	0.86 [0.34, 1.38]	0.88	Large	0.002
Interest in Nosocomial Knowledge	3.14 ± 0.65	3.81 ± 0.93	0.67 [0.22, 1.12]	0.79	Medium	0.004
Perceived Learning Burden*	2.29 ± 0.96	3.98 ± 0.86	1.69 [1.22, 2.17]	1.90	Large	< 0.001

All p-values remain significant after Bonferroni correction (alpha = 0.006). LBL = Lecture-Based Learning; BOPPPS-CBL = Bridge-in, Objective, Pre-assessment, Participatory Learning, Post-assessment, and Summary combined with Case-Based Learning. *Higher score indicates greater perceived burden (NOT reverse-coded). Cohen’s d interpreted as: small (0.2–0.5), medium (0.5–0.8), large (> 0.8)

Notably, the intervention group also reported significantly higher Perceived Learning Burden (3.98 ± 0.86 vs. 2.29 ± 0.96; mean difference = 1.69, 95% CI: 1.22–2.17, *p* < 0.001, Cohen’s d = 1.90), indicating increased cognitive effort associated with the active learning approach. We consistently describe this as perceived effort rather than confirmed increases in germane load. We acknowledge that we did not directly measure cognitive load types with validated instruments. This interpretation is speculative and requires further validation.

### Qualitative feedback

**Fig. 6:**
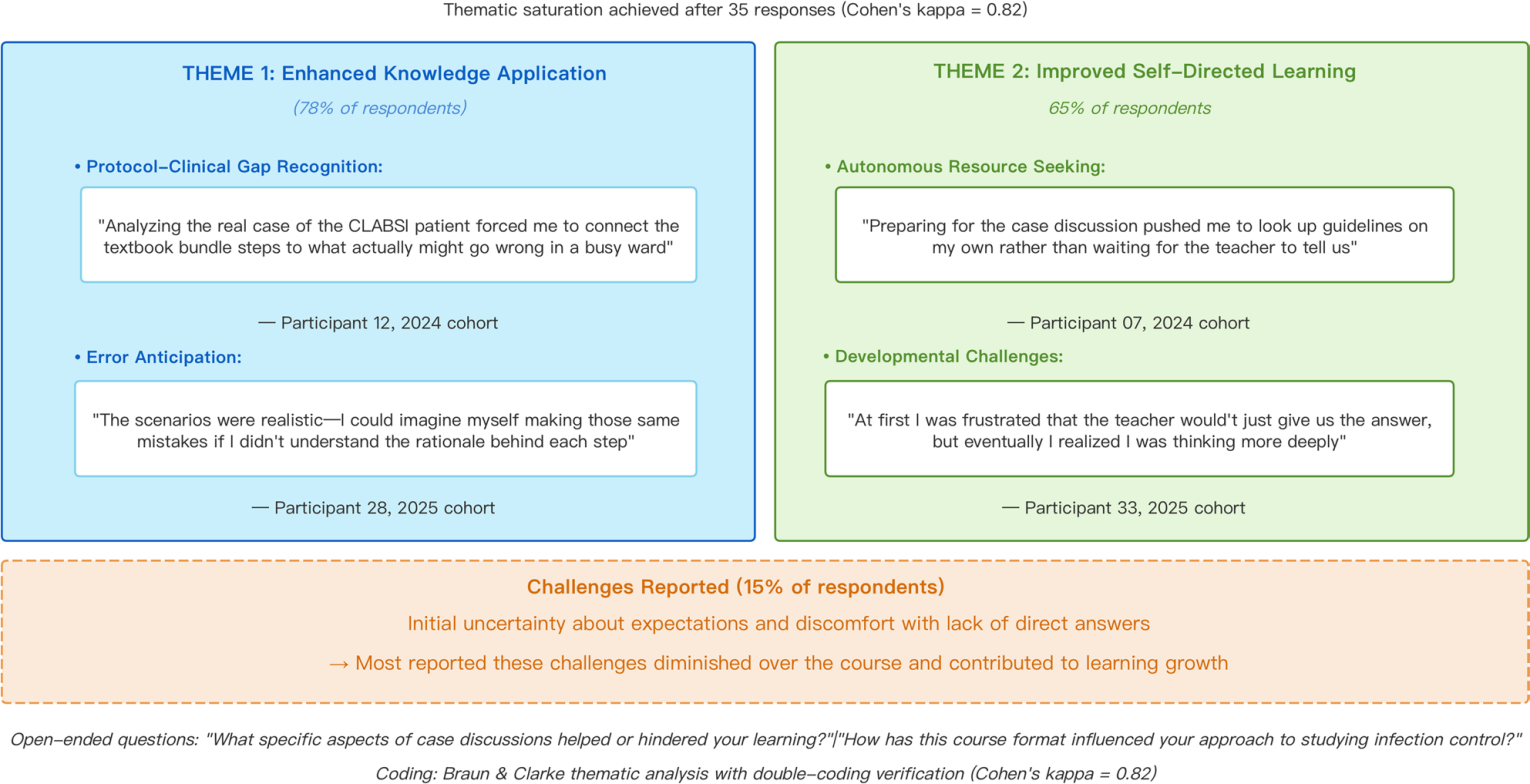
Key Themes Identified from Qualitative Feedback of BOPPPS-CBL Group Participants. Thematic map showing two superordinate themes-Enhanced Knowledge Application (34 of 43 respondents (78.1%)) and Improved Self-Directed Learning (65%)-with associated sub-themes and exemplar quotations. Inter-rater reliability: Cohen’s kappa = 0.82

Thematic analysis of open-ended responses from the BOPPPS-CBL group identified two superordinate themes: (Fig. 6)

Enhanced Knowledge Application (34 of 43 respondents (78.1%)): Students emphasized that case analysis facilitated the connection between theoretical protocols and clinical reality. Representative quotes included: ‘Analyzing the real case of the CLABSI patient forced me to connect the textbook bundle steps to what actually might go wrong in a busy ward’ (Participant 12, 2024 cohort) and ‘The scenarios were realistic-I could imagine myself making those same mistakes if I didn’t understand the rationale behind each step’ (Participant 28, 2025 cohort). Another student noted: ‘I now understand why each step in the bundle matters, not just what the steps are’ (Participant 15, 2024 cohort).

Improved Self-Directed Learning (28 of 43 respondents (65.1%)): Students reported increased autonomy in learning: ‘Preparing for the case discussion pushed me to look up guidelines on my own rather than waiting for the teacher to tell us’ (Participant 07, 2024 cohort). Several noted initial discomfort with the uncertain nature of case analysis, but recognized this as developmental: ‘At first I was frustrated that the teacher wouldn’t just give us the answer, but eventually I realized I was thinking more deeply’ (Participant 33, 2025 cohort). Another reflected: ‘I feel more confident researching topics independently now’ (Participant 22, 2025 cohort).

The 2023 control group received a standard course evaluation that did not include BOPPPS-specific open-ended questions; therefore, qualitative data were collected only from the 2024 and 2025 intervention cohorts. We acknowledge that qualitative data are only available for intervention cohorts, limiting direct comparison with the control group. These findings should be interpreted as a descriptive exploration of the intervention group’s experience rather than comparative evidence of the BOPPPS-CBL model’s superiority.

## Discussion

This multi-cohort quasi-experimental study suggests that the BOPPPS-CBL model is associated with improved HAI knowledge acquisition and positive learning engagement among undergraduate nursing students compared to traditional lecture-based instruction. The large effect size (partial eta-squared = 0.669) must be interpreted with extreme caution. As acknowledged in the Results section, this magnitude may be substantially inflated by the use of identical pre- and post-test instruments (testing effect), alignment between teaching content and assessment items (teaching-to-the-test), and the protocol-driven nature of HAI content that yields clear right/wrong answers. Therefore, the effect size should not be taken as a direct measure of pedagogical efficacy but rather as an upper-bound estimate under optimal testing conditions [22]. Given the quasi-experimental design with a historical control, we cannot infer a causal relationship between the BOPPPS-CBL model and the observed outcomes; the findings should be interpreted as associational evidence that requires confirmation in more rigorous designs (e.g., randomized controlled trials or interrupted time series). Additionally, the high effect sizes may reflect teaching-to-the-test effects or ceiling effects in the intervention group, where intensive preparation for case-based assessments led to optimized performance on the specific test items used. This is a critical distinction for readers attempting to replicate the model in different nursing contexts.

### Interpretation of knowledge gains

The adjusted mean difference of 13.58 points (95% CI: 11.14–16.03) favoring the BOPPPS-CBL group represents a robust educational gain. This improvement likely stems from the structured scaffolding provided by the BOPPPS framework combined with the contextualized application demanded by CBL. Unlike passive lecture-based instruction, the intervention required students to actively retrieve and apply knowledge during case analysis, a process known to enhance learning [23]. The consistency of effects across the 2024 and 2025 cohorts strengthens confidence in the intervention’s replicability.

Our findings align with and extend previous BOPPPS-CBL research in medical education [8–10]. However, the magnitude of improvement observed in our study exceeds that reported in some prior nursing education studies. This may reflect several context-specific factors: (1) the protocol-driven nature of HAI content, where clear ‘correct’ answers exist for bundle compliance, making the gap between theoretical knowledge and practical application particularly amenable to structured case analysis; (2) intensive small-group facilitation with high instructor-to-student ratios; (3) test alignment with learning objectives; (4) novelty effect of active learning; and (5) potential testing effects from identical pre/post assessments. These large effects may be specific to this context and may not generalize to all educational settings.

### Cognitive load considerations

The concurrence of higher perceived learning burden with higher satisfaction and learning outcomes aligns with theoretical frameworks in educational psychology [24, 25]. We speculate that the BOPPPS framework may have reduced extraneous cognitive load (via clear objectives, structured summaries, and chunked sessions) while optimizing productive cognitive engagement (via active case analysis requiring elaboration and schema construction).

However, we acknowledge important caveats: (1) we used a single-item measure of perceived burden rather than validated multi-dimensional cognitive load scales; (2) we did not directly measure different types of cognitive load; (3) the interpretation that burden represents ‘desirable difficulty’ is post-hoc and speculative. The increased perceived burden should be viewed as a trade-off of the active learning approach rather than an unqualified positive outcome. Future studies should employ validated instruments (e.g., NASA-TLX) to distinguish between germane and extraneous load.

### Limitations

Several limitations must be acknowledged when interpreting these findings: Collectively, these limitations threaten the internal and external validity of the study to a degree that precludes strong causal claims but does not negate the value of the preliminary, associational evidence provided.

Quasi-experimental Design: The use of a historical control group introduces significant threats to internal validity. History effects are particularly concerning-the COVID-19 pandemic transition during late 2022/early 2023 likely had profound impacts on nursing students’ baseline awareness, anxiety, and intrinsic motivation regarding infection control. The 2023 cohort (control) experienced the pandemic during their first year, while 2024/2025 cohorts (intervention) entered the post-pandemic era with potentially heightened infection control awareness independent of pedagogy. While we controlled for observed confounders and maintained instructional consistency, unmeasured differences between cohorts may have influenced outcomes. The single-institution setting further limits generalizability.Furthermore, this study lacked random assignment of students to conditions (historical control design) and complete blinding of instructors and assessors. Although graders were blinded to group assignment, instructors were necessarily unblinded, which may have introduced performance bias and detection bias.

Outcome Measurement: This study evaluated only Kirkpatrick Level 2 outcomes (learning and reaction) [13]. We did not assess long-term knowledge retention (> 3 months post-course) or the critical transfer of learning to clinical behavior (Kirkpatrick Level 3) during subsequent clinical rotations. Future research should employ longitudinal designs to assess whether these knowledge gains persist and translate into improved infection control practices. To bridge this gap in HAI education, we suggest that future researchers employ objective structured clinical examinations (OSCEs) with standardized patients or clinical audits during practicums to assess actual behavior change in clinical settings.

Assessment Tools: While the knowledge test demonstrated adequate psychometric properties, it focused on recall and application of protocols. It may not capture complex clinical reasoning or team-based competencies essential for HAI prevention in practice settings. The use of identical tests pre/post may introduce testing effects. The single-item measure of perceived learning burden lacks the precision of validated cognitive load instruments.

Qualitative Data Limitations: Open-ended questions about BOPPPS-CBL experience were only included in 2024/2025 evaluations, limiting qualitative comparability between groups. Thematic saturation assessment was limited by sample availability.

Resource Intensity: The BOPPPS-CBL model requires substantially more faculty resources than lecture-based instruction. While our discussion emphasizes theoretical explanations favorable to the BOPPPS-CBL model, alternative interpretations should be considered. The observed effects may reflect: (1) Hawthorne effects from the novel instructional approach; (2) increased study time and effort in the intervention group due to the more demanding format; (3) selection effects if students who persisted in the intervention group were more motivated; and (4) temporal confounding related to post-pandemic heightened awareness of infection control. These alternative explanations cannot be ruled out with the current design. We did not formally evaluate cost-effectiveness or faculty workload implications, which are crucial considerations for widespread adoption. Furthermore, we did not measure potential confounders such as out-of-class study time, individual learning strategies, or prior exposure to infection control content beyond the pre-test. These unmeasured variables may have contributed to the observed between-group differences and limit attribution of effects solely to the BOPPPS-CBL model. Regarding selection effects, although participation rates were high (> 91%) and the course was an elective, we cannot exclude the possibility that students with higher initial interest in infection control self-selected into the 2024/2025 cohorts. We did not measure intrinsic motivation or baseline attitudes toward infection prevention, which represents an unmeasured confounder.

### Practical implications

For educators considering BOPPPS-CBL implementation in infection control or other protocol-driven domains, we recommend several strategies based on our experience. First, phased implementation may help students adapt to active learning-consider introducing scaffolded case discussions gradually, beginning with simpler scenarios and progressing to complex, multi-factorial cases to manage cognitive load. Second, rigorous case development is essential-invest in authentic case development with content validity verification, ensuring cases include specific breach points amenable to root cause analysis. Third, faculty development is critical-train instructors in facilitative teaching techniques to transition from ‘sage on the stage’ to ‘guide on the side’ [26], emphasizing questioning over telling. Finally, balanced assessment approaches that include both individual accountability (post-assessments) and collaborative learning (group discussion) may optimize motivation and learning. Institutions adopting this model should recognize the specific context of this study (Chinese nursing education at a prestigious medical college) and consider how cultural and institutional factors may influence transferability. The high-stakes, protocol-driven nature of HAI education may make this domain particularly amenable to the BOPPPS-CBL approach, and effects may differ in more ambiguous or complex learning domains.Furthermore, given the substantial threats to internal validity (particularly the historical control design and the COVID-19 timing confound), we caution against direct replication of these effect sizes in other settings without first piloting the intervention with a contemporaneous control group. Any adoption of this model should include a rigorous local evaluation to account for context-specific confounders. This study did not include a formal cost-effectiveness analysis; the substantial faculty time required for case development and small-group facilitation (estimated 8 h of training plus 9 h of instruction per cohort) should be weighed against the observed learning gains before institutional adoption.

## Conclusions

Integrating the BOPPPS model with CBL appears to be a promising pedagogical strategy for undergraduate HAI education. This structured, active learning approach is associated with superior knowledge acquisition compared to traditional lectures and promotes engaged, self-directed learning. The associated increase in perceived cognitive load may represent productive cognitive engagement (increased perceived effort) when accompanied by improved outcomes, though this interpretation requires validation with dedicated cognitive load instruments.

Successful implementation requires careful attention to case design, faculty development in facilitative teaching, and recognition of the resource intensity inherent in small-group active learning. This model provides a potential blueprint for strengthening core competency education in nursing and other health professions where protocol adherence and clinical reasoning must coexist.

However, we emphasize that these findings are limited by the quasi-experimental design with historical controls. The large observed effects may reflect context-specific factors including post-pandemic heightened awareness of infection control. Longitudinal studies assessing knowledge retention and clinical behavior transfer (Kirkpatrick Levels 3–4) are essential to establish the true educational impact of this approach. Future research should employ objective structured clinical examinations or clinical audits during practicums to assess whether knowledge gains translate into improved clinical practice.

## Supplementary information

Supplementary Material 1: Supplementary Material 1: Supplementary Table S1: Mann-Whitney U test results for survey dimensions (non-parametric sensitivity analysis). Supplementary Table S2: Questionnaire items and factor loadings for the 8-dimension perception instrument.

## Data Availability

The datasets used and/or analyzed during the current study are available from the corresponding author on reasonable request. To protect participant anonymity, individual-level data will be shared only after removal of any potential identifying variables and with appropriate data use agreements.The study protocol, statistical analysis code, and de-identified data dictionary are available upon request.

## Abbreviations

ANCOVA: Analysis of Covariance
BOPPPS: Bridge-in, Objective, Pre-assessment, Participatory Learning, Post-assessment, and Summary
CBL: Case-Based Learning
CI: Confidence Interval
CLABSI: Central Line-Associated Bloodstream Infection
CAUTI: Catheter-Associated Urinary Tract Infection
CVI: Content Validity Index
EFA: Exploratory Factor Analysis
HAI: Healthcare-Associated Infection
I-CVI: Item-Level Content Validity Index
IRB: Institutional Review Board
KMO: Kaiser-Meyer-Olkin
LBL: Lecture-Based Learning
MCQ: Multiple Choice Question
MDRO: Multi-Drug Resistant Organism
S-CVI/Ave: Scale-Level Content Validity Index Average
SD: Standard Deviation
STROBE: Strengthening the Reporting of Observational Studies in Epidemiology
VAP: Ventilator-Associated Pneumonia
