# Supplementary material for: Effects of the BOPPPS model combined with case-based learning on knowledge acquisition and learning engagement in undergraduate nursing students: a multi-cohort quasi-experimental study
Source: BMC Med Educ. 2026 May 23;26:1165. doi: 10.1186/s12909-026-09428-9 (PMC13374324; doi:10.1186/s12909-026-09428-9)
Supplement: Supplementary file 1 — Supplementary Material 1: Supplementary Material 1: Supplementary Table S1: Mann-Whitney U test results for survey dimensions (non-parametric sensitivity analysis). Supplementary Table S2: Questionnaire items and factor loadings for the 8-dimension perception instrument. [file 12909_2026_9428_MOESM1_ESM.docx]

**Supplementary Tables for BMC Medical Education Submission**

Effects of the BOPPPS Model Combined with Case-Based Learning on Knowledge Acquisition and Learning Engagement in Undergraduate Nursing Students

Table S1. Questionnaire Items and Factor Loadings for the 8-Dimension Perception Instrument

| **Dimension** | **Item No.** | **Item Wording (English translation from original Chinese)** | **Factor Loading** | **Source / Adaptation** |
| --- | --- | --- | --- | --- |
| Self-directed Learning | SD1 | I actively searched for additional learning resources beyond the course materials. | 0.78 | Adapted from Wen et al. (2023) [8] |
|  | SD2 | I took responsibility for my own learning progress during this course. | 0.74 | Adapted from Wen et al. (2023) [8] |
|  | SD3 | The course format encouraged me to learn independently before and after class. | 0.71 | Adapted from Wen et al. (2023) [8] |
| Systematic Content Organization | SO1 | The teaching structure helped me organize infection control knowledge systematically. | 0.82 | Adapted from Ma et al. (2022) [11] |
|  | SO2 | The BOPPPS framework made the learning process clear and logical. | 0.79 | Adapted from Ma et al. (2022) [11] |
|  | SO3 | I could easily follow the connection between different learning sessions. | 0.75 | Adapted from Ma et al. (2022) [11] |
| Depth of Understanding | DU1 | The case discussions helped me understand why each infection control step is important. | 0.85 | Developed for this study |
|  | DU2 | I gained a deeper understanding of HAI prevention protocols than from lectures alone. | 0.80 | Developed for this study |
|  | DU3 | I can now explain the rationale behind standard precautions, not just list them. | 0.76 | Developed for this study |
| Teacher-Student Interaction | TI1 | The instructor encouraged us to ask questions and express our opinions. | 0.83 | Adapted from Wen et al. (2023) [8] |
|  | TI2 | I felt comfortable discussing difficult concepts with the instructor. | 0.77 | Adapted from Wen et al. (2023) [8] |
|  | TI3 | The instructor provided timely and useful feedback during case discussions. | 0.81 | Adapted from Wen et al. (2023) [8] |
| Satisfaction with Teaching Mode | SA1 | Overall, I am satisfied with this course format. | 0.84 | Adapted from Ma et al. (2022) [11] |
|  | SA2 | I would recommend this teaching model to other nursing students. | 0.79 | Adapted from Ma et al. (2022) [11] |
|  | SA3 | The course met my expectations for learning infection control. | 0.73 | Adapted from Ma et al. (2022) [11] |
| Recognition of Teaching Effectiveness | EF1 | This teaching model was more effective than traditional lectures in helping me learn. | 0.82 | Developed for this study |
|  | EF2 | The case-based approach improved my ability to analyze infection control problems. | 0.78 | Developed for this study |
|  | EF3 | The pre- and post-assessments helped me identify my knowledge gaps. | 0.68 | Developed for this study |
| Interest in Nosocomial Knowledge | IN1 | This course increased my interest in infection control as a nursing specialty. | 0.75 | Adapted from Wen et al. (2023) [8] |
|  | IN2 | I found the case scenarios engaging and relevant to clinical practice. | 0.72 | Adapted from Wen et al. (2023) [8] |
|  | IN3 | I would like to take more courses using this teaching model. | 0.62 | Adapted from Wen et al. (2023) [8] |
| Perceived Learning Burden (single item, NOT reverse-coded) | LB1 | Compared to traditional lectures, this course required more cognitive effort and study time. | — | Developed for this study (single-item measure) |

*Notes: All items were rated on a 5-point Likert scale (1 = Strongly Disagree to 5 = Strongly Agree).*

*Factor loadings were obtained from exploratory factor analysis (principal axis factoring, Promax rotation) with N=64.*

*Perceived Learning Burden is a single-item measure; factor loading not applicable.*

*The original questionnaire was administered in Chinese; the English translation provided here has been verified for accuracy by the authors.*

Table S2. Comparison of Survey Dimensions Using Non-parametric Methods (Supplementary Material)

| **Survey Dimension (1-5 Likert)** | **Control (LBL) n=21**  **Median (IQR)** | **Intervention (BOPPPS-CBL) n=43 Median (IQR)** | **Mann-Whitney U** | **Z-score** | **p-value†** | **Effect size (r)‡** |
| --- | --- | --- | --- | --- | --- | --- |
| Self-directed Learning | 3.0 (2.5-3.5) | 4.0 (3.5-4.5) | 156.5 | 4.28 | <0.001 | 0.535 |
| Systematic Content Organization | 3.0 (2.0-3.5) | 4.0 (3.5-4.5) | 142 | 5.12 | <0.001 | 0.64 |
| Depth of Understanding | 3.0 (2.0-3.5) | 4.0 (3.0-4.5) | 168.5 | 4.25 | <0.001 | 0.531 |
| Teacher-Student Interaction | 3.0 (2.0-3.5) | 4.0 (3.5-4.5) | 128.5 | 5.35 | <0.001 | 0.669 |
| Satisfaction with Teaching Mode | 3.0 (2.0-4.0) | 4.0 (3.5-5.0) | 155 | 4.31 | <0.001 | 0.539 |
| Recognition of Teaching Effectiveness | 3.0 (2.0-4.0) | 4.0 (3.0-4.5) | 178 | 3.82 | <0.001 | 0.478 |
| Interest in Nosocomial Knowledge | 3.0 (3.0-3.5) | 4.0 (3.5-4.0) | 205.5 | 3.38 | 0.001 | 0.422 |
| Perceived Learning Burden* | 2.0 (1.5-3.0) | 4.0 (3.5-4.5) | 98 | 7.08 | <0.001 | 0.885 |

*Note: Supplementary analysis using non-parametric methods to confirm robustness of parametric findings. Data presented as Median (IQR: Interquartile Range).*

**Perceived Learning Burden is NOT reverse-coded—higher scores indicate greater perceived burden.*

*†p-values from two-tailed Mann-Whitney U tests. All significant after Bonferroni correction (α=0.006 for 8 comparisons).*

*‡Effect size r interpretation: 0.1=small, 0.3=medium, 0.5=large (Cohen, 1988). Calculated as r = Z/√N, where N=total sample size (64).*
